# Pragmatic, Persistent, and Precarious: The Pathways of Three Minority Ethnic Women in STEM Higher Education

**DOI:** 10.1007/s10763-022-10337-8

**Published:** 2022-11-30

**Authors:** Billy Wong, Meggie Copsey-Blake

**Affiliations:** Institute of Education, University of Reading, London Road Campus, 4 Redlands Road, Reading, RG1 5EX UK

**Keywords:** Mathematics identity, Science identity, STEM identity, Women of color

## Abstract

Minority ethnic women are underrepresented in Science, Technology, Engineering, and Mathematics (STEM) higher education. Whilst existing studies, mostly in the US context, have provided valuable insights into racial and gender inequalities, there appears to be limited research in the UK. Through the lens of science identity, this article draws on qualitative data which was collected over three years, to appreciate how minority ethnic women develop their identity and belonging in STEM higher education, from the start to the end of their degrees. We present three case studies: (1) Nancy, a British East Asian Computer Science student, who appears *pragmatic* as she understands the extrinsic value of her degree, despite negative feelings and experiences; (2) Carol, a Black British Biomedical student, who can be seen as *persistent*, as supported by her intrinsic commitment to work in healthcare; and (3) Mawiya, a British Middle Eastern Mathematics student, whose experiences are somewhat *precarious,* because she must continuously negotiate her mathematics identity, which is often in question by herself and others. We discuss and compare the similarities and differences in the higher education pathways of these students. We also highlight the nuances of identity development and identity management, and consider multiple social inequalities for minority ethnic women. We conclude with a discussion of the implications of the findings for policy and practice.

## Introduction 

Research into the lived experiences of students can provide rich insights into the diverse and distinct ways in which individuals make sense of their education journey. In Science, Technology, Engineering, and Mathematics (STEM) education research, especially in the US context, there are growing recognitions that racial and ethnic minorities, particularly women, are underrepresented and minoritized, with unequal experiences and outcomes (e.g., Ong, Smith & Ko, 2018). Similar research is limited in the UK, where studies on issues of educational inequalities, particularly in STEM disciplines, are sparse and mostly absent from the literature.

Based on in-depth qualitative case studies, we explore patterns of student identity development and management in UK higher education, as reported by three minority ethnic women at different stages of their undergraduate STEM degrees. We consider how student experience can be shaped by multiple social inequalities, with a focus on the intersections of racism and sexism in higher education. We begin with *Nancy*, a Computer Science student, who we describe as *being pragmatic* because her choices and identities appear to be centered on the perceived exchange value of her degree. Next, *Carol*, a Biomedical student, who can be seen as *staying persistent*, as her intrinsic interests and aspirations to work in healthcare appear to have boosted her science identity, despite multiple barriers and social inequalities. Lastly, *Mawiya*, a Mathematics student, whose experiences can be interpreted as *feeling precarious,* as she expressed insecurity in the discipline and feelings of uncertainty about the future. We conclude with a discussion of the implications of our findings for policy and practice.

## Minority Ethnic Women in STEM Higher Education

Certain white affluent men are usually recognized as the global pioneers of Western science (Ong et al., 2018), but behind the scenes, women have always been pivotal to scientific discoveries and innovations. However, their contributions are often underacknowledged and women scientists are rarely mentioned or celebrated in the histories of STEM (Blackburn, 2017). More concerningly, there are considerably fewer representations of women from minority ethnic backgrounds, as the barriers to entry, participation, and attainment in STEM higher education remain unchallenged and underexplored in UK literature (AdvanceHE, 2020).

### Under-representation

International literature, especially from the USA, have explored the experiences of women and minority groups in STEM higher education (e.g., Carlone & Johnson, 2007; Rodriguez, Friedensen, Marron & Bartlette 2019), highlighting different factors that can contribute to their underrepresentation. These include chilly environments and unfair treatments (Wang & Degol, 2015), institutional inequalities and a lack of women or minority ethnic staff (Bottia, Stearns, Mickelson, Moller & Valentino, 2015), and social factors including familial aspirations and expectations (Lyon, Jafri & St. Louis, 2012). For instance, Ong (2005) found minority ethnic women in the physical sciences are often “in question” because they stand out from the norm, and must therefore compromise their social identities. These students may struggle to see themselves, or be perceived by others, as successful STEM students. As a result of racism, sexism, and other forms of discrimination, it seems inevitable that there are social barriers that come between minority ethnic women and their opportunities to develop an intelligible identity in STEM (Almukhambetova, Torrano & Nam, 2021; Nguyen & Riegle-Crumb, 2021).

### Identity

We view identity as socially constructed, multifaceted, temporal, and unfixed, as well as dependent on the participation of self and others (Butler, 1990; Gee, 2000). Whilst it is not possible to consider every facet of identity, we are particularly interested with how students negotiate their gender, racial, and ethnic identities, in order to manage their experiences of inequality, and to develop a disciplinary identity on their science degrees. The disciplinary identity in science, or *science identity*, is typically understood as how students recognize themselves and how they are recognized by others, in terms of their belonging, competence, and interest (Carlone & Johnson, 2007; Hazari, Chari, Potvin & Brewe 2020). Whilst there are other similar definitions of science identity (e.g., Avraamidou, 2020), we apply the notion of identity as our conceptual tool to unpack the identity work of students in their STEM degree journeys. Broadly, science identity encapsulates “a person’s understandings of self,” including how they “make sense of themselves and are made sense of by others,” with respect to the world of science (Kane, 2012, p. 458).

### Science as White Male-Dominated Endeavor

Science is often assumed as value-free and objective. However, critical feminist research suggests science is rooted in dominant colonial and patriarchal traditions that attribute greater power and control to white privileged men. This view on science suggests that the foundations of scientific knowledge are actually hierarchal and unequal (Keller, 1995; Tonso, 2006). These discourses often mean more challenging learning experiences or identity developments for underrepresented students, who must work harder to fit in a predominantly white male environment (Blackburn, 2017; Ong et al., 2018). This tends to legitimatize and privilege the patriarchy, whilst delegitimizing and overlooking the knowledges and contributions of women and ethnic minorities (Du, 2006). Therefore, within male-dominated environments such as STEM, women and minorities may have to consciously or unconsciously negotiate or assimilate their social identities, so not to confirm racist and gendered science stereotypes, and to gain a better sense of belonging (Keller, 1995). Other research perspectives suggest there is a cultural relevance to student subject choice, where culturally prescribed gender roles can influence students’ educational choices and career trajectories (Yazilitas, Svensson, de Vries & Saharso, 2013). However, girls and women are said to often demonstrate lower levels of confidence and self-efficacy in highly masculine and male-dominated fields (e.g., mathematics, physics), meaning they are often less likely to choose these STEM disciplines for study or career (MacPhee, Farro & Canetto, 2013). For girls and women from minority ethnic backgrounds, there are intersectional challenges from experiences of racism and the lack of visible role models in STEM (see below).

### Prior Research into Gender and Ethnicity in STEM Education

Existing research highlights a chilly climate for underrepresented groups in STEM higher education, especially for women who may often have to renegotiate their gender and disciplinary identities (Danielsson, 2012). Masculine connotations of the Physical sciences meant that the women consciously distanced themselves away from feminine gender expressions, in order to “do” Physics, and strengthen their disciplinary identities as student physicists.

A growing number of studies have also considered intersectionality: a critical feminist theory that aims to unpack the intersecting structures of inequality in society to better explain complex and dynamic experiences of oppression, especially the intersection of racism and sexism (Crenshaw, 1991). Avraamidou (2022) utilized the intersectional lens to explore the experiences of women undergraduate physics students from marginalized backgrounds in The Netherlands. The three case study participants told stories of otherness, persistence, hope and failure. Such discourses were said to highlight the kinds of identities that are socially accepted as well as those that are ostracized and excluded within the study of Physics. Avraamidou argues that disciplinary identity is strengthened through recognition, although this is fluid and, for different students, manifests not only as “explicit encouragement” but also as “no opposition” (p. 58). Avraamidou details that a physics identity is shaped by upbringing, including family, schoolteachers, university staff, students, and the local community, as well as being cultural dependent and influenced by inequalities and stereotypes.

Avraamidou’s (2020) earlier single case study of a young Muslim immigrant woman from Western Europe on a physics degree program also highlighted the intersecting influences of intrapersonal, interpersonal, and sociocultural factors. Various barriers were described in relation to religion and gender and their associated cultural expectations and negative stereotypes; as well as a general feeling of otherness due to different social inequalities, which can hinder feelings of belonging.

Gonsalves, Cavalcante, Sprowls & Iacono (2021) identified three undergraduate identity trajectories, narrated through stories of being in and out of school, and experiences of postsecondary science education contexts. These include the “expected trajectory,” the “persistent trajectory,” and the “new directions trajectory.” Students with an “expected trajectory” had access to resources which allowed for the accumulation of multiple dimensions of science-related capital, which encouraged them to see themselves as science people. Those who described a “persistent trajectory” drew more strongly on the narrative of science as a “natural choice” for them. However, they were persistent because they continued their science studies, despite not having the same passion for science as a child, and feeling more unsure about their pursuit of post-secondary science qualifications. Finally, students who were on a “new directions trajectory” demonstrated a gap between their expectations and the realities of post-secondary science education: some endured the gap, some gained new study strategies, and others compromised their identities in order to fit in. Gonsalves et al.’s article supports recent studies that highlight the influence of internal and external factors in how students understand and construct their identities and belongings in STEM degrees (e.g., Ferguson & Martin-Dunlop,  2021; Simpson & Bouhafa, 2020).

Opportunities such as co-operative education, work placements, and internships can shape how students engage with their STEM degrees (and other related disciplines). The success of cooperative education programs is mostly documented in the USA, and refers to tertiary education programs that are designed to prepare and diversify the STEM workforce, by providing students with opportunities to gain “real-world experience” working in a STEM industry whilst pursuing their field of study. Cooperative education programs, including work placements, and internships, are said to be linked to greater feelings of self-efficacy (Raelin, Bailey, Hamann, Pendleton, Reisberg & Whitman, 2014), higher financial returns, and greater employment security post-graduation (Wyonch, 2020). However, a recent study suggested that students who tend not to engage with STEM cooperative education programs, in both the USA and UK share similar attitudes and experiences of other real and perceived barriers, including anxiety about their abilities, the perceived cost of additional time to graduation, financial pressures, other paid work commitments, and clashes with family or social commitments (Ramirez, Smith, Smith, Berg, Strubel, Ohland & Main, 2016; Smith, Berg & Smith, 2015). The experiences and challenges of underrepresented students who are engaged in STEM higher education and its relative opportunities remains underexplored in the UK.

Other factors, such as that of parents and family members, can also influence the likelihood of students choosing and participating in STEM, from compulsory to tertiary education (Jones & Hamer, 2022). Cultural expectations and perceptions of success and financial security in particular disciplines and careers may vary between different social groups (Wong, 2016a). Similarly, access to opportunity in different subject areas may fluctuate depending on social contexts and inequalities, which is problematic considering minoritized students are often denied the same privileges as their white British counterparts (Bhopal, 2018), such as in STEM higher education.

In this article, we adopt the lens of intersectionality to unpack the identity trajectories of three minority ethnic women in UK STEM higher education. More specifically, we explore how these students manage and negotiate their gender and racial identities throughout their degree study.

## The Study

This study utilized the intersectionality lens to unpack the identity trajectories of three undergraduate minority ethnic women in UK STEM higher education. Minority ethnic students in UK higher education are proportionally better represented in STEM (26.9%) than in non-STEM (17.4%) degrees (AdvanceHE, 2020), with around 25% of all UK-domiciled university students being identified as from a minority ethnic background. Whilst there is growing awareness and research in UK higher education on structural racism and inequalities, there are also concerns and recognitions that the experiences, trajectories and outcomes of minority ethnic students in STEM education are more challenging and difficult than their White British counterparts (Wong, 2016b; Wong, ElMorally & Copsey-Blake, 2021a). This article offers a qualitative and longitudinal study into the STEM degree journeys (Year 1, Year 2, Year 3) of three minority ethnic women, with the aim to document and detail their identity development and trajectory over the course of their undergraduate degree. It explored how these students manage and negotiate their gender and racial identities throughout their degree. These participants were part of a larger study which broadly focused on the experiences of minority ethnic students in higher education (see below, and Wong, Copsey-Blake & ElMorally, 2022).

### University Context and Recruitment of Participants

The study was situated in a medium-sized English university with a student size and composition that broadly reflects the national population (between 10,000–20,000 students). The case study institution is neither extreme nor atypical in terms of student diversity and outcome, with a spread of STEM and non-STEM departments. After ethics approval, data collection for this 3-year study began in 2018 with a call for STEM undergraduate participants, especially those who self-identify as a minority ethnic student. The recruitment call included messages in students’ virtual learning environment and short in-person presentations of the project aims. Whilst the target was UK-domiciled minority ethnic undergraduates, we also had interest from students who self-identified as White British. A total of 88 in-depth interviews and 155 journal reflections were collected over the three years from 57 unique students in the full project, with 43 from minority ethnic backgrounds (of which, 32 were women).

The project recruited students at different stages of their undergraduate degree, some students participated for three years, whilst others for less. Our three case study students were selected as typical cases of minority ethnic women, each exemplifying a specific theme of STEM experience and journey (see below: pragmatic, persistent and precarious). Nancy, a British East Asian Computer Science student, participated for three years (3 interviews, 6 reflections, 2018–2021); Carol, a Black British Biomedical student, for two years (2 interviews, 4 reflections, 2018–2020); and Mawiya, a British Middle Eastern Mathematics student, for four years, as she also took part in the pilot interview study (4 interviews, 6 reflections, 2018–2021). Whilst the data for each case varied, the data for these students were rich and illustrative of those with similar viewpoints, including a longitudinal perspective on their identity development and negotiations. This longitudinal data was not available for most other minority ethnic women who participated in the study.

### Data Collection

Participation in the research involved submission of two guided journal reflections and an interview each year, over the course of their degree. The journal reflections required the students to submit their responses electronically at the end of the autumn (December) and summer (June) terms. A template was sent to students with guided questions, which asked for their written views and reflections about their summer experiences and plans, the beginning and end of term, including transition into university for first-year participants, academic progress, available support, and overall thoughts on their development in the last few months. A typical journal submission was around 700–1000 words.

The semi-structured interviews were conducted in the spring terms (January–March), between the journal reflections. On average, these lasted an hour and were conducted in quiet rooms across the university in Year 1 of the project, and these were moved online in years 2 and 3 of the study, due to the coronavirus pandemic. The quality and depth of data collected seemed consistent between online and face-to-face interviews, although we acknowledge there is an experiential difference between in-person and virtual data collection (see Archibald, Ambagtsheer, Casey & Lawless, 2019). Students were asked to share their views on a range of topics, including their choice of STEM degree study, experiences of university, and the role of “race” and ethnicity in their STEM education. For instance, we asked students to reflect on how their ethnicity may have shaped their university experiences, followed by specific questions about their thoughts and even experiences, if any, of racism. To build rapport and trust, all interviews had an extra hour scheduled for participants to feel more comfortable and learn about the research and researcher. Whilst conversations about race and racism can be uncomfortable and requires sensitivity, our reflection is that our students appear open and sincere in their own views and perspectives. We also reassured participants that there are no right or wrong answers and their identities would be anonymized. All interviews were audio recorded and transcribed verbatim, with sensitive details removed. An e-voucher was provided as a token of appreciation.

### Data Analysis

Data analysis was informed by a social constructionist perspective, which understands social phenomena as socially constructed and discursively produced. Interview transcripts and journal reflections were imported into the qualitative data analysis software, NVivo. Provisional codes were created, and themes and concepts were later refined and expanded as we moved back and forth between the data and analyses in an iterative process (Corbin & Strauss, 2014). A coding framework was established, with a guided list of definitions for each code, after the authors independently coded the interview transcripts and journal reflections by relevant themes. These themes were then discussed and compared, with any differences on the application of codes debated until a consensus was reached. The codes were then grouped into higher-level themes to appreciate the lived experiences of minority ethnic students, with a focus on their perspectives at different points in their higher education journey. The key themes included reflections of their academic and social lives, especially their challenges and opportunities, as well as their aspirations for the future.

Below, we present the experiences of each student under overarching themes, namely as pragmatic, persistent and precarious. These themes are not mutually exclusive but would encapsulate the essence of their STEM higher education journey. Our three cases provide a more focused understanding of how minority ethnic women experience and manage their identities in different stages of their degree education. We bring together their similarities and differences in the discussion section through a cross-case synthesis (Yin, 2017), highlighting the key issues and potential implications.

## Nancy: Being Pragmatic

Nancy is a British East Asian woman who studies Computer Science, a technology degree with a history of male and masculine stereotypes. She considers herself to be middle-class with two university-educated parents. Her father works in pharmaceutical sales and her mother is a stay-at-home parent, previously an accountant.

Consistent with UK national figures, around one in five of Nancy’s classmates are women (81.6% of computer science undergraduates are men, AdvanceHE, 2020). Although minority ethnic groups are slightly better represented in computer science than in other STEM degrees (28.5% vs 25.6% for all STEM, AdvanceHE, 2020), most students are still men which means minority ethnic women remain very few in numbers.

Nancy chose computer science because she considered it to have potential in terms of employment security, career prospects and future financial returns, suggesting her pragmatic approach to her degree choice. Popular amongst East Asian families, Nancy admitted that her parents were influential in her educational journey, with the good intention to ensure that her choices are “right.” For higher education, this meant a degree with a clearer and safer future career pathway (Wong, 2016b). Although Nancy was passionate about English literature, she believed there would be less job opportunities in the arts, humanities, and the social sciences, especially compared to STEM industries (DeWitt, Archer & Moote, 2019).

In the first year of undergraduate study, Nancy said she initially struggled with a sense of belonging and did not find friends who shared her values and interests. She said, “I have found it quite difficult to get accustomed to university life… I found that in terms of accommodation, people can be much more difficult to deal with than expected.” Her social struggle prompted Nancy to question her degree choice and she even considered dropping out or changing course, although she was worried about how her parents would react and so she persevered (Jones & Hamer, 2022; Reilly & Hurem, 2021).

However, Nancy experienced and witnessed casual racism and racial microaggression, often from peers in and around her student accommodation and social circle. She recalled being called “a Chinese bitch” by intoxicated flatmates who “banged on my door at 2am,” as well as being mocked for her appearance and unfairly accused as a supporter of dog meat consumption. Reflecting on her first year, Nancy said she has learned “how and when to keep quiet even when I have been wronged,” highlighting the normalization of racial injustice and the disempowerment that minority ethnic students can feel due to racism.

In the second year, Nancy’s social life improved as she established a closer friendship group, who happened to be mostly students from minority ethnic backgrounds, although Nancy insisted that was not deliberate. However, during the pandemic, halfway into her degree, people perceived to be of East Asian origins, including Nancy, were subject to heightened levels of racism (UK Parliament, 2020), from verbal attacks in the wider public to microaggression from fellow peers. For example, Nancy recalled at the beginning of one lecture, “they were playing this coronavirus song today… on the main computer screen… but it was sung in a very stereotypical Chinese accent. And I just didn’t find it funny.” Although Nancy was unsure if this act was directed at her, she “just turned it off” and said, “it did kind of irritate me.” Racism and racial microaggression, as demonstrated in the behaviors of Nancy’s academic peers, exemplify how racial inequalities are typically normalized and accepted in university spaces. For underrepresented students such as Nancy, this can undermine their feelings of belonging as well as their identity development in higher education.

In the third year, Nancy opted to do a year-long work placement which was confined to working from home. After securing her offer, Nancy recalled a dismissive comment from a male peer, who downplayed her success by adding that “you’re able to find a placement quite easy because you’re a girl.” Another male student also joked to Nancy that girls can use their “feminine charms to get answers… [or preferable treatments] in the workspace and stuff.” According to Nancy, some “people actually see it [being a woman] more as an advantage” because the tech sector, as well as other disciplines engaged in cooperative education programs (Wyonch, 2020), appear to be on a mission to increase diversity and improve representations of women in STEM.

Here, the accomplishments of women and minorities can be challenged by others, which can manifest into self-doubt. Nancy said she had to convince herself that “I couldn’t have been rubbish… because otherwise they wouldn’t accept me whatever gender I am, but definitely, I think other students have an opinion on that.” The apparent need to justify her success, even if only to herself rather than to her peers, highlights the deep-rooted nature of male and masculine dominance in computer science, and more broadly, the impact of underrepresentation on minority ethnic women and their sense of belonging in STEM.

On the experiences she gained from the work placement, Nancy appears overwhelmed by the high standards of people she saw in employment, and felt inadequate and unprepared. Her taste of the profession seems to have shaken her development of a professional identity in STEM, despite being on track to achieve a “first-class” degree (i.e., the highest band).

Reflecting on her degree, Nancy said a turning point for her was when she must apply for work placements as part of her course, because it “forced me to properly consider my future career.” Nancy shared that she now aspires to work in investment banking and admitted her hesitance about a computer science-related career, asserting that the work requires “highly intelligent” individuals. Nancy is due to return to university for her final year.

## Carol: Staying Persistent

Carol identifies herself as a Black British woman, who studies Biomedical Sciences, which was a backup option as she was unsuccessful in her bid to do a degree in medicine. She described her family as working-class. Her mother attended university and is a secondary school teacher, whilst her father stopped after secondary education and currently does office work in the local council. Carol mentioned her family has had, and continues to have, financial struggles.

Women are equally represented in the biological sciences (49% nationally), although minority ethnic students are low in numbers (just over 20% nationally, AdvanceHE, 2020). Carol was unsure of the actual numbers in her cohort but confirmed that “there’s definitely way, way, way more Caucasians than ethnic minorities.”

She describes herself as active and adventurous, constantly “trying new things… meeting new people,” akin to a social butterfly. For instance, she has taken an optional module in British Sign Language and tried out new and different sports. Carol said she enjoys “developing friendships… and getting to know people on deeper levels.” However, Carol said that she struggled to find friendship within her first-year student accommodation, as her flatmates were mostly White British. Despite “making the effort,” she found “they were a bit passive aggressive and cold” and concluded that her flatmates were “closed minded… about somebody outside of their own culture.” Nonetheless, Carol persevered and thrived socially as she found solace in the wider student community. Carol admitted, however, that she does not feel like herself in academic conversations, as she tries to “mimic the social standard… around certain people” to feel heard or acknowledged, and to maintain an intelligible and intellectual STEM identity amongst her majority peers.

Carol shared an episode of her interaction with a member of staff during her second year which, like Mawiya below, also illustrates her precarious identity in STEM. In a segment of an assignment, Carol was graded a zero, which baffled her because she thought “it was something that I actually done correct.” Carol said she emailed her tutor “to complain about that and she just never replied.” However frustrated, because Carol “didn’t want to escalate the issue,” she let it go and explained that “I kind of don’t want to be the one to cause trouble.” Here, her consciousness of not wanting to be seen as a potential troublemaker may reflect wider and often negative stereotypes about Black women (McGee & Bentley, 2017). Although Carol said her encounters of racism, sexism, and microaggressions are limited and often perceived as trivial at university, such as “making fun of my hair,” she added, “I may be a bit oblivious… to these things.” Carol suggested she was raised to be self-assured in that “if I tried my best, that’s all that really matters…[and] if I do badly, I just need to try a bit better.” Here, Carol appears persistent and seems to have developed a level of resilience against different setbacks, including implicit racism. However, on the above episode, she felt wronged and disempowered. The silver lining is that the zero grade had limited impact on her overall outcome. Reflecting on her undergraduate study, Carol believes she has become “more resilient” and “more confident,” and remains positive that she is “equipped with the skills I need to handle” issues of racism and sexism in the future. To her credit, by the end of her undergraduate, Carol was accepted onto a postgraduate program that provides a pathway into the medical profession.

## Mawiya: Feeling Precarious

Mawiya is a British Middle Eastern woman who studies Mathematics. She was unsure about her socioeconomic background but stated both her parents went to university. Her father is a software engineer and her stepmother works in early years education. She was also our pilot participant, which meant we interviewed her from the first year to the fourth and final year, as she undertook a year-long work placement.

Nationally, just under four in 10 students are women in undergraduate mathematics (37.2%) and one in five from a minority ethnic background (24.3%, AdvanceHE, 2020). According to Mawiya, there is just a handful of minority ethnic women in her cohort, and she is “the only person wearing a hijab,” which makes her rather conscious of her appearance in large settings such as lectures. Interestingly, Mawiya does not believe her university experiences are negatively affected by her ethnicity or gender, although she admitted that “I rarely think about” these issues. She did acknowledge, however, that “every now and again,” the topic of race “comes up but… I just don’t care.” Here, Mawiya appears unaware or perhaps despondent to inequalities stemmed by ethnicity and gender (Wong, ElMorally, Copsey-Blake, Highwood & Singarayer, 2021b).

For Mawiya, mathematics is an intriguing subject because it “actually makes sense to me” and she found it “really easy” in school. She continued mathematics at university because “I don’t know what else I wanna do” but she recognized that a mathematics degree will provide her with broad career options. Mawiya described feeling anxious and unprepared about her transition from school to university. She also struggled in the first year to find commonality with her White British flatmates due to their incessant drinking, partying and late-night activities and noises, which she said had caused her sleep deprivation. As the year progressed, alongside the departure of her “inconsiderate flatmates,” Mawiya began to find her sense of belonging and formed social groups with students from her mathematics degree, where “we hang out all day” and she even “arranged a cinema trip.” Seemingly by choice, most of her friends were minority ethnic women, which Mawiya explained is because “we have a lot of things in common.” Socially, Mawiya seems content about her peer groups and engagement with the wider university community.

Academically, Mawiya started positively with strong, first-class grades. However, in the second year, she struggled with motivation and confidence, recalling that “from the first day of term, I felt very overwhelmed and even though I wasn’t, I felt very behind.” Her anxieties grew and she “couldn’t focus on studying and I started falling behind.” Whilst tutor support was available, Mawiya admitted she “never emailed or asked question outside of tutorials [because] I’m too anxious to ask,” which meant her struggles were unattended. She eventually rediscovered her motivation, with the support of peers, and passed the year with slightly lower grades, an “upper second-class.”

In her third year, Mawiya undertook a yearlong industrial placement and worked in a healthcare company as a statistician. Her role involves data monitoring and statistical analysis, which was “very relevant to my degree.” Mawiya was initially full of praise because she “really enjoyed” the work experience and the breadth of skills that she was developing. However, she later reflected that “the more I think about it, I feel like the more negative I think of it,” which seems to have fed into her precarious identity in mathematics. Here, Mawiya experienced self-doubt and questioned her own skills and abilities. As she recalled:

**I feel like I’m not progressing and not even meeting expectations… just felt really slow sometimes… It’s just feels like everyone else is sitting there concentrating and actually able to do some work and again, I am not.**

Whilst her peers, who completed a similar work experience, shared their joys and satisfactions, Mawiya asked, “why am I the only one who is feeling really shit about this?”. Her struggle with motivation continued into the fourth and final year, which seems to recur at the start of each academic year. As she analyzed, “it is like a low mood, or seasonal affective disorder.” Alongside on and off lockdown rules and social distancing due to the coronavirus pandemic, Mawiya found it difficult to engage with online learning, which added to her mental stress and anxiety. She even lowered her expectations and reassured herself that “all right, let’s just aim for a 2:2 [lower second-class],” which is another band lower than her second-year grades. Mawiya’s identity in mathematics appears fragile as she contemplated retaking the year, or taking a year out, “possibly travelling and still working somewhere.” She remains uncertain of her career pathway and admitted that “I haven’t even really figured out… honestly it’s all in the air now.” More worryingly, Mawiya described experiencing an overall deterioration in her mental (and physical) wellbeing throughout her degree.

Overall, Mawiya’s higher education has been a journey of uncertainty. Her identity in mathematics started off strongly, reaffirmed by high grades, but she struggled in subsequent years and began to self-doubt her ability. She often felt overwhelmed and underprepared, which impacted her motivation and materialized into lower outcomes. Her work experience was valuable, but her observation of others and their apparent competency, also undermined her self-efficacy and appeared to weaken her mathematics identity (Carlone & Johnson, 2007; Hazari et al., 2020). Whilst Mawiya remains unsure and uncertain about the future, she did offer an optimistic view in that “the university experience, whatever the grades you get… along the way, you actually get closer to thinking about what you want to do with your life.”

## Discussion & Conclusion

This article explored the STEM degree journeys of three minority ethnic women. Through the cases of Nancy, Carol and Mawiya, we presented three pathways and relationships with STEM higher education that detailed how UK minority ethnic women experienced STEM education at different stages of their undergraduate degree. Below, we draw comparisons from our findings of our three case studies and discuss the meanings and implications of the three pathways: *being pragmatic, staying persistent*, and *feeling precarious*.

## The three pathways

To some extent, being pragmatic and having an (intrinsic or extrinsic) interest in STEM were shared by all three women in their choices of study. For students such as Nancy, a pragmatic approach towards STEM study reflects an awareness of its exchange value, especially in the job market. For Nancy, her study of computer science was primarily driven by what she, and her family, agreed as a “safer” degree choice for future employment (Craig, Verma, Stokes, Evans & Abrol, 2018). Nancy described herself as being competent and interested in the discipline, but her decision appears to have been strategically informed, which suggests her participation in a computer science degree is highly extrinsic (Wong, 2016a).

The second pathway was comparably different. The example of Carol, who we described as “staying persistent,” reflects the experiences of students whose STEM education journeys are mostly driven by their intrinsic interests and aspirations in the discipline. For Carol, her passion and persistence in biomedicine, especially as a future healthcare professional, seems to have given her added strength and resilience in her chosen pathway, particularly when faced with social inequalities (Gonsalves et al., 2021). Her passion and determination to realize her goal meant she could embody a strong sense of belief and self-recognition in her discipline, which forms a key aspect of her STEM identity (Carlone & Johnson, 2007; Hazari et al., 2020). Whilst pragmatic and persistent students are both goal-oriented, Nancy seems to be driven by extrinsic motivations, and Carol by intrinsic motivations. However, both extrinsic and intrinsic approaches seem to strengthen their visions of anticipated outcomes, which can act as a booster for personal confidence or resilience, as and when needed.

For students who are less clear about future goals, their STEM identities may be more ambiguous, which we described in our third pathway as “feeling precarious.” Through the case of Mawiya, we show how students can choose to study for a mathematics (or STEM) degree without any preparation for their long-term goals. Their choices can be driven by intrinsic as well as extrinsic motivations. Mawiya started positively, achieving high grades, but after the first year, her identity in mathematics withered, as she struggled with motivation, anxiety and confidence, lowering her outcomes (Núñez-Peña, Suárez-Pellicioni & Bono, 2013).

The experiences of Nancy, Carol, and Mawiya have highlighted pragmatic, persistent, and precarious journeys in STEM higher education. All three women reported shared experiences of collective struggles, especially during their transitions to university and in the beginning stages of their degrees. There are often few, if any, fellow minority ethnic women in their discipline of study, which can impact their science identity development and sense of belonging in STEM disciplines. All three women suggested they had experienced compatibility issues with White peers in both academic and social contexts, including during lectures and in student accommodation (Avraamidou, 2020), and reported a greater sense of belonging when they formed friendship groups with other minority ethnic women, considered to be more likeminded.

### Implicit Racism and Sexism

Of particular concern is the prevalence of subtle forms of racism and sexism, in and out of the education context, and the impact of microaggressive behaviors on underrepresented students’ sense of belonging in higher education. Our case study students all mentioned problems with their white flatmates and academic peers, with implicit and explicit lived experiences of racial microaggression. On reflection, Nancy felt her encounters with racism and sexism may have unconsciously undermined her identity in computer science, as well as in higher education and the workplace (see Ong et al., 2018). Being pragmatically driven, Nancy’s identity in computer science and STEM appears less as a concern, as long as she has faith in the exchange value of her degree after graduation. Here, the goal-orientated approach of a pragmatist may have dampened the impact of racism and sexism in Nancy’s STEM journey. However, her career intentions, which lead away from computer science and STEM, suggests that minority ethnic women such as Nancy are unlikely to pursue a career in STEM (British Science Association [BSA], 2020).

Similarly, subtle racism seems to have undermined Carol’s identity and belonging in higher education, as she recalled her conscious efforts to “mimic the social standard” in academic spaces. Whilst all students can mimic behaviors to gain social acceptance, the “social standard” in an academic space, especially in STEM higher education, is typically racialized and gendered as white and male dominated. As a Black woman, Carol appears highly conscious of her positionality but was readily adept to present and perform herself in a way that she recognized would be valued and accepted in STEM higher education (McGee & Bentley, 2017). Here, Carol actively engaged in identity work as part of her socialization to “fit in,” as she attempted to adopt established STEM discourses and practices (Ferguson & Martin-Dunlop, 2021; Ong,  2005). Although Carol may have learnt to navigate the social rules of STEM higher education, the responsibility here is still firmly on the minoritized individual to manage and modify their own identity, which unfortunately means existing structures and practices, including sexist or racist discourses, remain prevalent and unchallenged. Consistent with the current literature, these discourses often mean more challenging learning experiences or identity developments for underrepresented students, who must work harder to fit in within a predominantly white male environment (Blackburn, 2017; Du, 2006; Ong et al., 2018).

Such challenges seem to persist for underrepresented students during cooperative education programs and work placements or internships. Albeit in unique ways, Nancy and Mawiya expressed insecurities about their abilities and belongings in their relative STEM disciplines and work contexts. For instance, Nancy experienced dismissive remarks made by a male peer on her offer of a work internship, with accusations of positive discrimination that, Nancy suggested, undermined credit to her own abilities and successes. In turn, Nancy felt the need to justify her success of being offered a place. Underrepresented students such as Nancy therefore face challenges in managing internalized feelings of self-doubt in order to develop an intelligible STEM identity (Carlone & Johnson, 2007; Hazari et al., 2020; Kane, 2012).

Comparably, Mawiya’s year of industrial placement provided her with real-life work experience in the use of statistics, which is often regarded as a positive, because it fosters students’ development of a professional identity (Jackson,  2017). However, Mawiya ended up feeling more discontent and uncertain about her abilities in mathematics (MacPhee et al., 2013). Here, Mawiya compared herself with others, including active STEM professionals, but felt inadequate and underprepared (Avraamidou, 2020). Although Mawiya confessed her naivety on issues of sexism and racism, and did not make explicit references to these social inequalities in her work experiences, it was notable that her self-efficacy and identity in mathematics were weaker after her internship as she even contemplated halting her degree (MacPhee et al., 2013; Raelin et al., 2014). Like Nancy and Carol, a longitudinal study of Mawiya’s degree journey has provided us with a unique insight into the fluidity of her science identity development, from avidity in the first year to apathy in the final year (Avraamidou, 2020, 2022). In short, Mawiya’s journey in STEM is comparatively precarious. She was uncertain about her future, including in mathematics, a discipline which once provided her with intrinsic as well as extrinsic motivations.

As Bowen (2020) warned, the existence of gender and racial bias within an organization can have negative impacts on the experiences of women in work placements, especially implicit discourses, and gender and racial microaggressions. As we have seen in the cases of Nancy and Miwaya, this has implications for programs that are designed to diversify the STEM workforce, as well as other related disciplines, and ease the transition for students, from education to the labor market (Wyonch, 2020). Despite their subtleties, the emotional and mental cost of microaggressive behaviors on the higher education experiences and identities of underrepresented students is apparent. Here, the message for policy and practice is that there must be an ongoing need and commitment from universities and staff to review, reflect and challenge existing practices and discourses in STEM departments, especially implicit and microaggressive practices and ideologies underpinned by gender and ethnic stereotypes (Blackburn, 2017; Museus, Palmer, Davis & Maramba, 2011). A clear strategy should be developed that highlights the different steps needed to address these inequalities, with active involvement from senior leaders, subject tutors, professional staff, and most importantly, students.

### Challenging Systemic Structures and Inequalities

We recognize that breaking down STEM stereotypes requires a collective effort, beyond the university, especially with the perceptions from the media and wider society (Ward & Grower, 2020). However, as educational institutions, it is imperative that inequalities of ethnicity and gender are recognized and resisted within STEM higher education. According to McGee (2020), structural inequalities in STEM must be exposed and dismantled, and we must move away from an individualistic lens that frame minorities as the problem that requires “fixing,” so to speak. As Archer, Godec, Barton, Dawson, Mau and Patel (2021) argued, it is the field of STEM that requires a critical examination of its structures and practices, with meaningful and impactful changes dependent on a long-term commitment to disrupt and equalize existing structures of inequality. The first step, therefore, is to acknowledge and recognize the complexity of these social barriers, followed by a review and implementation of strategies and policies that aim to break down these obstacles. Different approaches will be needed at different levels and contexts, but such aspirations will only have mileage if sufficiently supported by university leaders and senior management (Universities UK [UUK], 2020).

In the teaching and learning context, STEM staff must be able to facilitate a diverse and inclusive curriculum and environment (Ceglie, 2021). Dewsbury (2017) argued the mindset of staff ought to shift from one that focuses on the deficits of students to one that acknowledges the role of program structures, departmental practices, and the wider university in shaping student experiences and outcomes. In the classroom, Dewsbury urged that the diverse identities and backgrounds of STEM students should be actively acknowledged and appreciated, which may include a greater use of dialogic pedagogy that encourages broader participation and perspectives.

We want to recognize that there is now greater awareness and work by higher education staff and institutions to promote diverse practices (e.g., University College London [UCL], 2020). However, Winberg, Adendorff, Bozalek, Conana, Pallitt, Wolff, Olsson et al. (2019) found limited evidence of such work within the STEM disciplines. Haynes and Patton (2019) explained that staff in STEM, especially those from White ethnic backgrounds, tend to view their disciplines as “race-neutral,” drawing on the positivist and objective paradigm. For instance, the argument here may be that the contents of their teaching are characterized by facts and formulas, which does not change or discriminate, regardless of the tutor or the student. Yet, the ways in which these knowledges are produced or disseminated are not always critiqued or reflected, and these are still dominated by a male Eurocentric and colonial lens. Perhaps the prerequisite of a diverse and inclusive STEM curriculum ought to begin with staff and their acknowledgement of potential racial and gender biases within their discipline.

Our study has shed light on the consciousness as well as cautiousness of minority ethnic women in their approach to STEM education, at different stages of the degree. The examples of Nancy, Carol, and Mawiya add to the UK literature on STEM higher education experiences, especially with a longitudinal focus on underrepresented students. We believe it is important to make sense of lived experiences, and recognize the breadth and depth of how different factors and inequalities can shape current and future identities in STEM. In sum, it seems evident that more time, awareness and attention needs to be given to the experiences and challenges of minority ethnic women students, as a result of racism and sexism in higher education. These inequalities and injustices appear to be heightened in highly racialized and gendered environments such as STEM degrees, and the onus must not continue to be upon minority ethnic women to navigate these challenges individually. More research is needed on the unique (but often racialized and gendered) journeys of underrepresented students if social justice and mobility through STEM higher education is to be realized.

## References

[CR1] AdvanceHE (2020). *Equality and higher education - Student statistical report 2020*. AdvanceHE.

[CR2] Almukhambetova, A., Torrano, D. H. & Nam, A. (2021). Fixing the leaky pipeline for talented women in STEM. *International Journal of Science and Mathematics Education*. 10.1007/s10763-021-10239-1

[CR3] Archer, L., Godec, S., Barton, A. C., Dawson, E., Mau, A. & Patel, U. (2021). Changing the field: A Bourdieusian analysis of educational practices that support equitable outcomes among minoritized youth on two informal science learning programs. *Science Education,**105*(1), 166–203. 10.1002/sce.21602 10.1002/sce.21602 PMC8453532 34588711

[CR4] Archibald, M. M., Ambagtsheer, R. C., Casey, M. G., & Lawless, M. (2019). Using zoom videoconferencing for qualitative data collection: Perceptions and experience of researchers and participants. *International Journal of Qualitative Methods, 18,* 1609406919874596. 10.1177/1609406919874596

[CR5] Avraamidou L (2020). “I am a young immigrant woman doing physics and on top of that I am Muslim”: Identities, intersections, and negotiations. Journal of Research in Science Teaching.

[CR6] Avraamidou L (2021). Identities in/out of physics and the politics of recognition. Journal of Research in Science Teaching.

[CR7] Bhopal, K. (2018).* White privilege: The myth of a post-raccial society*. Bristol University Press.

[CR8] Blackburn H (2017). The status of women in STEM in higher education: A review of the literature 2007–2017. Science & Technology Libraries.

[CR9] Bottia, M., Stearns, E., Mickelson, R., Moller, S. & Valentino, L. (2015). Growing the roots of STEM majors: Female math and science high school faculty and the participation of students in STEM. *Economics of Education Review, 45*, 14–27.10.1016/j.econedurev.2015.01.002

[CR10] Bowen T (2020). Examining students’ perspectives on gender bias in their work-integrated learning placements. Higher Education Research & Development.

[CR11] British Science Association [BSA] (2020). *The state of thesector: Diversity and representation in STEM industries in the UK*. Author.

[CR12] Butler J (1990). Gender trouble.

[CR13] Carlone HB, Johnson A (2007). Understanding the science experiences of successful women of color: Science identity as an analytic lens. Journal of Research in Science Teaching.

[CR14] Ceglie R (2021). Science faculty’s support for underrepresented students: Building science capital. International Journal of Science and Mathematics Education.

[CR15] Corbin, J., & Strauss, A. (2014). *Basics of qualitative research: Techniques and procedures for developing grounded theory (4th ed.)*. Sage

[CR16] Craig, C. J., Verma, R., Stokes, D., Evans, P. & Abrol, B. (2018). The influence of parents on undergraduate and graduate students’ entering the STEM disciplines and STEM careers. *International Journal of Science Education,**40*(6), 621–643. 10.1080/09500693.2018.1431853

[CR17] Crenshaw K (1991). Mapping the margins: Intersectionality, identity politics, and violence against women of color. Stanford Law Review.

[CR18] Danielsson AT (2012). Exploring woman university physics students ‘doing gender’ and ‘doing physics’. Gender and Education.

[CR19] DeWitt, J., Archer, L. & Moote, J. (2019). 15/16-year-old students’ reasons for choosing and not choosing physics at Alevel. *International Journal of Science and Mathematics Education,**17*(6), 1071–1087. 10.1007/s10763-018-9900-4

[CR20] Dewsbury, B. M. (2017). On faculty development of STEM inclusive teaching practices. *FEMS Microbiology Letters*, 364(fnx179). 10.1093/femsle/fnx179 10.1093/femsle/fnx179 28922842

[CR21] Du X-Y (2006). Gendered practices of constructing an engineering identity in a problem-based learning environment. European Journal of Engineering Education.

[CR22] Ferguson, D. & Martin-Dunlop, C. (2021). Uncovering stories of resilience among successful African American women in STEM. *Cultural Studies of Science Education, 16, *461–484. 10.1007/s11422-020-10006-8

[CR23] Gee JP (2000). Identity as an analytic lens for research in education. Review of Research in Education.

[CR24] Gonsalves, A. J., Cavalcante, A. S., Sprowls, E. D. & Iacono, H. (2021). “Anybody can do science if they’re brave enough”: Understanding the role of science capital in science majors’ identity trajectories into and through postsecondary science. *Journal of Research in Science Teaching, 58*(8), 1117–1151. 10.1002/tea.21695

[CR25] Haynes, C. & Patton, L. D. (2019). From racial resistance to racial consciousness: Engaging white STEM faculty in pedagogical transformation. *Journal of Cases in Educational Leadership,**22*(2), 85–98. 10.1177/2F1555458919829845

[CR26] Hazari, Z., Chari, D., Potvin, G. & Brewe, E. (2020). The context dependence of physics identity: Examining the role of performance/competence, recognition, interest, and sense of belonging for lower and upper female physics undergraduates. *Journal of Research in Science Teaching,**57*(10), 1583–1607. 10.1002/tea.21644

[CR27] Jackson D (2017). Developing pre-professional identity in undergraduates through work-integrated learning. Higher Education.

[CR28] Jones KL, Hamer JJ (2022). Examining the relationship between parent/carer’s attitudes beliefs and their child’s future participation in physics. International Journal of Science Education.

[CR29] Kane, J. M. (2012). Young African American children constructing academic and disciplnary identities in an urban science classroom. *Science Education, 96*(3), 457–487. 10.1002/sce.20483

[CR30] Keller EF (1995). Reflections on gender and science.

[CR31] Lyon, G. H., Jafri, J. & St. Louis, K. (2012). Beyond the Pipeline: STEM Pathways for Youth Development. *After School Matters*, *16*, 48–57. URL: https://www.niost.org/2012-Fall/beyond-the-pipeline-stem-pathways-for-youth-development

[CR32] MachPhee, D., Farro, S., & Canetto, S. S. (2013). Academic self-efficacy and performance of underepresented STEM majors: Gender, ethnic, and social class patterns. *Analyses of Social Issues and Public Policy, 13*(1), 347–369. 10.1111/asap.12033

[CR33] McGee EO (2020). Interrogating structural racism in STEM higher education. Educational Researcher.

[CR34] McGee, E. O. & Bentley, L. (2017). The troubled success of Black women in STEM. *Cognition and Instruction,**35*(4), 265–289. 10.1080/07370008.2017.1355211

[CR35] Museus SD, Palmer RT, Davis RJ, Maramba DC (2011). Racial and ethnic minority students’ success in STEM education.

[CR36] Nguyen, U. & Riegle-Crumb, C. (2021). Who is a scientist? The relationship between counter-stereotypical beliefs about scientists and the STEM major intentions of Black and Latinx male and female students. *International Journal of STEM Education, 8*, Article 28. 10.1186/s40594-021-00288-x 10.1186/s40594-021-00288-x PMC10857866 38343634

[CR37] Núñez-Peña, M. I., Suárez-Pellicioni, M. & Bono, R. (2013). Effects of math anxiety on student success in higher education. *International Journal of Educational Research,**58*, 36–43. 10.1016/j.ijer.2012.12.004

[CR38] Ong M (2005). Body projects of young women of color in physics: Intersections of gender, race, and science. Social Problems.

[CR39] Ong, M., Smith, J. M. & Ko, L. T. (2018). Counterspaces for women of color in STEM higher education: Marginal and central spaces for persistence and success. *Journal of Research in Science Teaching,**55*(2), 206–245. 10.1002/tea.21417

[CR40] UK Parliament. (2020). Chinese and East Asian communities: Racism during Covid-19. Volume 682: Debated on Tuesday 13 October 2020. URL: https://hansard.parliament.uk/Commons/2020-10-13/debates/858E78B5-1049-4480-A1A7-5362FC12F47E/ChineseAndEastAsianCommunitiesRacismDuringCovid-19

[CR41] Raelin, J. A., Bailey, M. B., Hamann, J., Pendleton, L. K., Reisberg, R. & Whitman, D. L. (2014). The gendered effect of cooperative education, contextual support, and self-efficacy on undergraduate retention. *Journal of Engineering Education,**103*, 599–624. 10.1002/jee.20060

[CR42] Ramirez, N., Smith, S., Smith, C., Berg, T., Strubel, B., Ohland, M. & Main, J. (2016). From interest to decision: A comparative exploration of student attitudes and pathways to co-op programs in the United States and the United Kingdom. *International Journal of Engineering Education*, 32(5A), 1867–1878. URL: http://www.ijee.ie/contents/c320516A.html

[CR43] Reilly, D. & Hurem, A. (2021). Designing equitable STEM education: Guidelines for parents, educators, and policy-makers to reduce gender/racial achievement gaps. 10.13140/RG.2.2.24828.46723

[CR44] Rodriguez, S., Friedensen, R., Marron, T. & Bartlett, M. (2019). Latina undergraduate students in STEM: The role of religious beliefs and STEM identity. *Journal of College and Character,**20*(1), 25–46. 10.1080/2194587x.2018.1559198

[CR45] Smith, S., Berg, T. & Smith, C. (2015). *Making co-op work: An exploration of student attitudes to co-op programs*. Paper presented at the IEEE Frontiers in Education Conference (FIE). 10.1109/FIE.2015.7344173

[CR46] Tonso KL (2006). Student engineers and engineer identity: Campus engineer identities as figured world. Culture of Science Education..

[CR47] University College London [UCL] (2020). *BAME Awarding Gap Project: Staff toolkit 2020*. Author.

[CR48] Universities UK [UUK] (2020). *Tackling racial harassment in higher education*. Author.

[CR49] Wang, M-T., & Degol, J. (2015). School climate: A review of the construct, measurement, and impact on student outcomes. *Educational Psychology Review, 28*, 315–352. 10.1007/s10648-015-9319-1

[CR50] Ward LM, Grower P (2020). Media and the development of gender role stereotypes. Annual Review of Developmental Psychology.

[CR51] Winberg, C., Adendorff, H., Bozalek, V., Conana, H., Pallitt, N., Wolff, K., Olsson, T. & Roxå, T. (2019). Learning to teach STEM disciplines in higher education: A critical review of the literature. *Teaching in Higher Education,**24*(8), 930–947. 10.1080/13562517.2018.1517735

[CR52] Wyonch, R. (2020). Work-ready graduates: The role of co-op programs in labour market success. *C.D. Howe Institute Commentary 562*. 10.2139/ssrn.3520206

[CR53] Yazilitas, D., Svensson, J., de Vries, G. & Saharso, S. (2013). Gendered study choice: a literature review A review of theory and research into the unequal representation of male and female students in mathematics, science and technology. *Educational Research and Evaluation,**19*(6), 525–545. 10.1080/13803611.2013.803931

[CR54] Yin RK (2017). Case study research and applications: Design and methods (6th edition).

[CR55] Wong Billy (2016). Minority Ethnic Students and Science Participation: a Qualitative Mapping of Achievement, Aspiration, Interest and Capital. Research in Science Education.

[CR56] Wong, B. (2016b). *Science education, career aspirations and minority ethnic students*. Palgrave MacMillan.

[CR57] Wong, B., ElMorally, R., & Copsey-Blake, M. (2021a). ‘Fair and square’: What do students think about the ethnicity degree awarding gap?. *Journal of Further and Higher Education, 45*(8), 1147–1167. 10.1080/0309877X.2021.1932773

[CR58] Wong, B., ElMorally, R., Copsey-Blake, M., Highwood, E., & Singarayer, J. (2021b). Is race still relevant? Student perceptions and experiences of racism in higher education. *Cambridge Journal of Education, 51*(3), 359–375. 10.1080/0305764X.2020.1831441

[CR59] Wong B, Copsey-Blake M, ElMorally R (2022). Silent or silenced? Minority ethnic students and the battle against racism. Cambridge Journal of Education.

